# Biopharmaceutical Financialization and Public Funding of Medical Countermeasures (MCMs) in Canada During the COVID-19 Pandemic

**DOI:** 10.34172/ijhpm.2023.6936

**Published:** 2023-05-27

**Authors:** Ipek Eren Vural, Matthew Herder, Agnieszka Doll, Janice E. Graham

**Affiliations:** ^1^Department of Political Science & Public Administration, Middle East Technical University, Ankara, Turkey; ^2^Department of Political Science, Dalhousie University, Halifax, NS, Canada; ^3^Faculty of Health Sciences, Simon Fraser University, Burnaby, BC, Canada; ^4^Health Law Institute, Dalhousie University, Halifax, NS, Canada; ^5^Department of Pharmacology, Dalhousie University, Halifax, NS, Canada; ^6^Department of History and Sociology, University of British Columbia Okanagan, BC, Canada; ^7^Department of Pediatrics, Dalhousie University, Halifax, NS, Canada; ^8^Department of Sociology and Social Anthropology, Dalhousie University, Halifax, NS, Canada

**Keywords:** Transparency, Intellectual Property, Access Policies, Biomanufacturing, Innovation, Vaccines

## Abstract

**Background:** Analysing the Canadian government’s efforts to support the development of COVID-19 "medical countermeasures" (MCMs), this article seeks insights into political economy as a driver of pandemic response. We explore whether Canadian public funding policy during the pandemic involved departures from established practices of financialisation in biopharmaceutical research and development (R&D), including the dominance of private sector involvement in an intellectual property (IP) intensive approach to innovation underscoring profit, and governance opacity.

**Methods:** We interrogate public funding for MCMs by analyzing how much the Government of Canada (GoC) spent, how those funds were allocated, on what terms, and to whom. We identify the funding institutions, and the funds awarded between February 10, 2020, and March 31, 2021, to support the research, development, and manufacturing of MCMs, including diagnostics, vaccines, therapeutics, and information about clinical management and virus transmission. To collect these data, we conducted searches on the Internet, public data repositories, and filed several requests under the *Access to Information Act (1985)*. Subsequently, we carried out a document-based analysis of electronically accessible research contracts, proposals, grant calls, and policy announcements.

**Results:** The GoC announced CAD$ 1.4 billion for research, development and manufacturing of COVID-19 MCMs. Fully 68% (CAD$ 959 million) of the announced public funding was channelled to investment in private sector firms. Canadian public funding showed a consistent focus on early and late stage development of COVID-19 MCMs and the expansion of biopharmaceutical manufacturing capacity. Assessing whether Canada’s investments into developing COVID-19 MCMs safeguard affordable and transparent access to the products of publicly funded research, we found that access policies on IP management, sharing of clinical data, affordability and availability were not systematic, consistent, or transparent, and few, if any, mechanisms ensured long-term sustainability.

**Conclusion:** Beyond incremental change in policy goals, such as public investment in domestic biomanufacturing, the features of Canadian public policies endorsing financialization in the biopharmaceutical sector remained largely unchanged during the pandemic.

## Background

**Key Messages**

**Implications for policy makers**

- Lack of transparency in the purpose, conditions, and decision-making procedures of public funding prevents recognition of the government’s efforts in building up current and future pandemic preparedness in Canada.
- Public funding of innovation (research, development and manufacturing of COVID-19 medical countermeasures [MCMs]) is dominated by market-based policies that endorse the primacy of private sector involvement and profit.
- Pro-public safeguards attached to publicly funded research grants and contracts remained lax.
- Intellectual property (IP) management of public funding neglects balancing exclusivities with requirements that would allow public access to innovative products.
- Commitments towards data transparency remained too broad to ensure the protection of public health and further scientific inquiry.

**Implications for the public**

Financialized capitalism, which emerged as the dominant growth model in the 1970s, spurred shortages of medical countermeasures (MCMs) during the pandemic. Governments in Canada and beyond deployed billions of dollars of public funding to mobilize research, development, and manufacturing of COVID-19 MCMs. We explore whether the Canadian government’s investments in biomedical COVID-19 interventions safeguarded affordable and transparent access to the resulting products. Our findings show access policies on intellectual property (IP) management, sharing of clinical data; affordability and availability have been far from systematic, consistent, or transparent.

Government responses to crises can perpetuate or change the prevailing political economy, ie, global relations of production and attendant economic, political, and ideological practices.^1-3^ The public health and economic crises spurred by the COVID-19 pandemic were expected to mark shifts in the prevalent global political economy since the 1970s, that is, financialized capitalism.^4^ In defiance of fiscal conservatism endorsed by neoliberal policies, governments and central banks responded to the joint pandemic and economic crisis with expansionist monetary policies. Shortfalls in the global supply of COVID-19 diagnostics, therapeutics, and vaccines, what we refer to here as medical countermeasures (MCMs) were linked to developments spurred by financialized capitalism, such as the globalization of production and the strengthening of intellectual property (IP) protection.^5-7^ Industrial policies involving public investments in the manufacturing of strategic goods and sectors, including MCMs, regained favour.^8,9^ Like many of its G7 counterparts, the Canadian government aimed to provide rapid access to emerging COVID-19 MCMs by spending billions of dollars in research, development, manufacturing, procurement, and delivery.^10^ Governments everywhere expressed commitment to equitable and affordable access to products of publicly funded research.^11,12^ Several measures were proposed across high-, middle- and low-income countries, including the development of patent or product pools that allow sharing of data and IP to develop and manufacture MCMs^13^; the incorporation of pro-public safeguards to enhance accessibility, transparency and affordability of the resulting products^14^; and, legislative measures allowing access to medical technologies without IP holders’ consent. In Canada, the *Emergency Response Act* passed by Parliament in March 2020 included a patent override for COVID-19 related patented inventions.^15^ Whether government efforts to combat the COVID-19 pandemic will mark a shift or a deepening of “financialized capitalism” in the long term remains to be seen.

 To gain insights about the current political economy as a driver of COVID-19 pandemic response, we analyse Canadian federal government efforts to support the development of MCMs. Priorities and practices underpinning public funding policy are explored, including how their allocation affects knowledge production processes, accessibility and transparency of the MCMs. In theory, Canada’s spending on MCMs as well as other programs and services intended to curb COVID-19 without a concomitant concern over the country’s future fiscal well-being that underpinned neoliberalism’s diminution of the state and reliance upon the market, could portend political economy change. Given global health interests, we inquire whether the Canadian governments’ commitments to funding MCMs marks a shift from established practices of financialization. More specifically, we explore if public funding: (*i*) promotes access to research outputs, (*ii*) balances IP intensive knowledge production with public safeguards, and (*iii*) improves the transparency of decision-making processes. Focussing on the Canadian experience, our research aims to contribute to a growing body of international literature about the implications of public funding of COVID-19 MCMs for innovation, protection of public health and democratic accountability.^6,16-19^

 We begin with a review of the basic features of financialization, its established practices, and some of the access challenges that those practices engendered during the pandemic.

###  Financialization, Innovation Policy and Shortages of COVID-19 Medical Countermeasures 

 Norms and practices associated with financialization, — the dominant growth model in contemporary capitalism since the 1970s —, spurred shortages of COVID-19 MCMs during the pandemic. By financialization we refer to the increased control over investment resources by capital market actors (such as institutional investors, hedge funds, private equity and venture capital firms) that began in the 1970s, and since then has come to define a number of norms and practices under the rubric of global capitalism.^20,21^ Financialization assigns priority to enhancing short-term profits over longer-term socially beneficial and sustainable outcomes,^22-26^ spreading neoliberal ideas and governance models,^27^ strengthening IP exclusivities^28-30^; and, replacing local manufacturing capabilities with global supply chains.^31,32^

 Financialization is well advanced in the biopharmaceutical industry, entrenched in the practices that structure the research, production, and commercialization of MCMs. The expansion of capital market actors, specifically venture capital and private equity firms — investments in biotechnology and pharmaceutical companies since the 2000s, has transformed business models everywhere. Companies, especially in liberal market economies^33^ such as the United States, the United Kingdom, Canada, Australia, New Zealand, and Ireland, have become oriented towards financial markets and prioritized shareholder values often at the expense of investments in research and development (R&D) as well as of localized production.^34-36^ Financialized business models^34^ endorsed cost efficiency to increase shareholder value, and drove the organization of just-in-time production networks by transnational firms, steering the globalization of production.^5,37-39^ Various stages of domestic manufacturing capabilities were dispersed across boundaries to benefit from low wage economies, R&D externalities, and jurisdictions with lower taxes.^31^ In many countries, previously public manufacturing plants have been not only privatized, but integrated into the global supply chains, while their productive role and functions were redefined. Inevitably, the formation of global supply chains involved the erosion of local manufacturing capacities across many geographies. Connaught Laboratories in Toronto, for example, which secured biologics sufficiency in Canada and abroad throughout most of the last century, was bought by Mérieux (now Sanofi Pasteur) in the 1980s, and its vaccine manufacturing capacity was repurposed in line with Sanofi’s global production priorities.^40^

 Public policies that govern the biopharmaceutical industry in Canada and beyond have endorsed these patterns of financialization.^41^ Innovation policies that advocate maximizing private sector profits and attracting funding from institutional investors have been adopted to foster product development and growth in the biopharmaceuticals sector.^42,43^ Three policy instruments have been deployed to sustain these “market based innovation policies.”^44^

 First, public funding has been used to attract investments by financial market actors, such as venture capital and private equity firms. Since 2013, the Canadian federal government has invested more than CAD$ 800 million into venture capital funds to derisk “innovative” Canadian start-ups.^43,45-47^ Generous leveraging from public investment helped Canadian venture capital reach record heights of CAD$ 6.2B in 2019.^48^ Biopharmaceuticals (broadly corresponding to the health and life sciences category in Canadian Venture Capital and Private Equity Association data) have emerged as the second highest recipient of venture capital investments in Canada.

 Second, public funding policies increasingly endorsed an IP intensive approach to knowledge production. Compared with the era of welfare capitalism (1945-1979), the goal of public funding allocated to support upstream research in scientific processes has been transformed. Rather than generating public goods available to all, public funding informed by market based innovation policies encouraged universities to commercialize research outputs by transforming research knowledge into IP, with a view to structuring and expanding collaborations with the private sector.^44,49^ Public funding of scientific research in the United States and Canada has thus been used to support the development of assets for biopharmaceutical firms to commercialize^35,50^ and venture capitalists to invest.^41,43,51,52^ University-industry collaborations and public private partnerships have become the preferred governance models for capital transfers made through public funding.^53^ In Canada, the University of Toronto’s Medical and Related Services was established to partner with the private sector to commercialize research, receiving large sums of provincial and federal public transfers.^54^

 Third, in addition to using IP to collaborate with industry and commercialize research, nation-states’ expansion and enhanced enforcement of IP rights, which include patent rights, trade secrets, and a variety of sources of exclusivity that have been integrated into the regulatory system, marked a important corollary of financialised capitalism both before and during the pandemic.^5,27,39^ Stronger IP protection has been a precondition of the globalization of biopharmaceutical production. Powerful financial and business lobbies stood behind the strengthening of a global IP regime during the 1980s and 1990s, which crystallized with the enactment of the Trade Related Aspects of Intellectual Property Rights (TRIPs) Agreement in 1994.^28-30^ Global enforcement of patent protection and trademarks ensured that the transnational pharmaceutical industry could spread production worldwide without compromising the ownership of their technology.^38^ Stronger IP exclusivities are central to maximizing financial investors’ shareholder profits in biopharmaceutical companies not only through higher prices from marketed products, but also through expediting the profits derived from the sale of intangible assets that are still in the research pipeline.^5,39^

 An important corollary of financialization, including in biopharmaceuticals, has been the growing centralization and opacity of regulatory decision-making.^55,56^ Transparency of regulatory processes in the biopharmaceutical sector is essential to secure public health, drug safety, and efficacy.^57^ Concerns over standards, mismanagement of pharmaceutical safety and efficacy evidence, conflicts of interests in biopharmaceutical regulation,^58^ rising drug prices despite extensive public funding of their development^36,59^ have led to a greater need for transparency. While governments in Canada and globally responded with measures to make more information available about pharmaceutical evidence, regulatory reviews, pricing, and public funding, tensions have prevailed over how to incorporate these measures into practice.^58^

###  Access Challenges During the COVID-19 Pandemic

 The above features of financialization, especially the globalization of production, the attendant hollowing out of local manufacturing capabilities, and stronger IP exclusivities, generated global shortages of COVID-19 vaccines and other MCMs during the pandemic. Although industry claimed that public funding and IP mediated profit incentives were necessary for the development of COVID-19 MCMs,^60^ exclusivities significantly restricted scaling up of production and trade of COVID-19 MCMS during the pandemic.^5,6^

 Canada’s vaccine rollout was delayed because it lacked manufacturing capacity adaptable for the production of mRNA vaccines, the leading COVID-19 vaccine platform.^61,62^ In the face of urgent worldwide demand for life-saving MCMs, Canada remained reliant on Indian and European pharmaceutical transnationals to secure supplies.^63^ Yet in the absence of domestic manufacturing capacity, trade proved to be an imperfect way to gain access to MCMs. Like other high-income countries, Canada rushed to make exclusive bilateral deals with transnational vaccine and therapeutics developers. Although Canada secured the highest number of COVID-19 vaccine doses per capita in the world, reserving enough to vaccinate its population five times over,^64^ production glitches during the scaling up of manufacturing, and export restrictions imposed by other nation-states (competing to secure earlier and faster access of their citizens) caused delays in delivery schedules, which translated into a global access crisis in early 2021.

 As large transnationals organize research, development and manufacturing of biopharmaceuticals globally, Canadian public funding policy inevitably affects and has been affected by power struggles at the international level to control knowledge, technology, development and procurement of COVID-19 MCMs. An IP intensive approach to knowledge production, endorsed by financialization, crippled global solidarity mechanisms put in place to organize equitable product development, manufacture and distribution. The launch of the World Health Organization (WHO) COVID-19 Technology Access Pool (CTAP), and the United Nations Technology Access Partnership in May 2020 was intended to enhance sharing of knowledge and know-how through non-exclusive, royalty free licenses, and voluntary non-enforcement of IP rights.^65^ Promoting open science to accelerate innovation and scale up of manufacturing globally,^65,66^ CTAP constituted an important challenge to IP intensive knowledge production endorsed by financialization. Yet, global firms controlling most exclusive IP rights showed no interest in participating in such platforms.^67^ Without their collaboration, would-be producers lacked the covert know-how to manufacture COVID-19 MCMs. Governments of most high-income countries, including Canada, did not endorse open science solutions proposed by CTAP.^68^ Lack of support for CTAP during this early stage of the pandemic not only reinforced the IP intensive approach to innovation and production but also ensured that mechanisms that maintain the industry’s control over MCM-related IP, such as the COVID-19 ACT (Access to COVID-19 Tools) Accelerator and its vaccines initiative, COVID-19 Vaccines Global Access (COVAX),^19^ formally housed by the WHO and jointly overseen by the GAVI Vaccine Alliance and Coalition for Epidemic Preparedness Initiative, remained the only alternative to accelerate access to emerging products. Initially organized as a procurement and financing tool, COVAX, aimed to subsidize vaccine doses for low-income countries through donations and sales to high-income countries, philanthropy by the private sector, and COVAX’s financial instrument, the International Finance Facility for Immunization.^69^ This multilateral effort, however, failed to attract sufficient timely donations from high-income countries and private sector corporations to deliver MCMs to pandemic struck low-income countries. In an attempt to diversify their supply sources, high-income countries purchased directly from multinational developers doses of COVID-19 MCMs that exceeded by several times the amount needed to treat their populations. Compounded by limited global supply, such hoarding of MCMs by high-income countries^70,71^ augmented the shortages, and affordability problems for low-income countries, leaving millions of people unprotected against the pandemic.^72^ Advance Market Commitment tool used by COVAX that obliged it to race with high-income countries to sign bilateral vaccine deals with a handful of suppliers^70^ not only intensified competition in a supply restricted global vaccine market, but also left the organization far behind in its bid to provide vaccine supplies to low-income countries.^73^

 When multilateral efforts through ACT Accelerator and its COVAX initiative remained underfunded,^74^ a group of developing countries turned towards exercising the public health safeguards in the World Trade Organization (WTO) agreement to expand their access to COVID-19 MCMs. The October 2020 WTO application by the Indian and South African governments to waive TRIPs provisions to allow them to expand access to COVID-19 MCMs was blocked until May 2021^75^ due to financial and business lobby opposition in concert with governments of some (if not all) advanced economies. Although negotiations for the IP waiver were initiated in the TRIPs Council, following partial support from the US government for a vaccine waiver,^76^ the process was slow and opposed by powerful lobbies, reflected in an European Union (EU) counter proposal in June 2021 that suggested TRIPs compliant use of compulsory licensing instead of waivers.^77^

 It remains uncertain whether ongoing struggles for access to MCMs will generate political will at national and international levels to better balance innovation and access. Given the Canadian government’s commitments, the amount of public funding involved, and high public interest in outcomes, it is important to investigate how much the Canadian federal government spent, how those funds were allocated, on what terms, and to whom.

## Methods

 In analysing the federal government’s funding toward COVID-19 MCMs, we sought to comprehensively capture: (*i*) investments made by one or more federal government departments to support research, development, manufacturing or procurement of diagnostics, vaccines, and therapeutics; and (*ii*) research grants awarded between February 10, 2020 and March 31, 2021 to develop COVID-19 MCMs and produce information about clinical management and transmission (Table 1). Federal procurement of personal protective equipment and other supplies was excluded from our analysis to zero in on MCMs that require substantial research before use, such as diagnostic technologies (eg, rapid COVID-19 tests), therapeutics, and vaccines. Independent grants and funding calls issued by provincial governments, private agencies and foundations, and non-governmental organizations were also excluded from the scope of this analysis. Provincial governments’ contributions were only included in the analysis when these participated in joint calls with the Federal Government, such as with Canadian Institutes of Health Research (CIHR) or Genome Canada grants.

**Table 1:** Institutional Channels of Public Funding Announced by the Canadian Government

**Channels of Funding**	**Type of Funding**	**Recipient**	**$ (Milion)**	**Date Announced**	**Duration**	**Purpose**
CIHR, NSERC, Genome Canada^a^, & Provincial Research Grant Organizations	R&D	144 Grant projects funded	124.0	10/02/2020, 19/03/2020, 31/03/2020, 19/06/2020, 23/04/2020	1-2 Years, depending on project	COVID-19 rapid response and rapid research calls.
CIHR	R&D	CATCO trial	3.6	04/2020	NA	To advance the Canadian arm of WHO Solidarity Trial, investigating safety & efficacy of medications in improving COVID-19 mortality.
CIHR	R&D	Canadian International Border Study/McMaster Health Laboratories	2.5	10/2020	NA	To advance research on benefits and risks of airport based COVID-19 testing, and public health measures for travellers.
CIHR	R&D	17 Projects	9.3	12/3/2021	NA	Emerging COVID-19 research gaps and priorities.
CIHR	R&D	COVID-19 Variants Network	14.3	26/03/2021	NA	To support new research on COVID-19 variants and establish a network to align variants research.
CIHR	R&D	Canadian Network of COVID-19 Trials	6.0	20/01/2021	NA	To expand existing clinical trial networks to coordinate research on COVID-19 tools.
NSERC Alliance &Tri-agency Applied Research Rapid Response to COVID-19	R&D	317 Grant projects funded	15.7	05/08/2020	NA	To stimulate collaborations between university researchers, public, non-profit sectors, and industry.
Genome Canada	R&D	NA	1.5	2/04/2020	NA	To support genomics-informed solutions to COVID-19.
Federal Government	R&D	CanCOGeN	40.0	23/04/2020	NA	To coordinate a COVID-19 viral and host genome sequencing effort across Canada.
SIF	R&D and BioMan	AbCellera & Medicago	192.0	23/03/2020	NA	To advance antibody therapy and vaccine research, and construction facility.
SIF	R&D and BioMan	NA	600.0	23/04/2020	2 Years	Direct support to Canadian companies for large scale projects.
Canada Foundation for Innovation	R&D	VIDO-InterVac	11.0	23/03/2020	NA	To support operating costs through to March 2023.
Western Economic Diversification	BioMan	VIDO-InterVac	12.0	23/03/2020	NA	To develop and upgrade a vaccine manufacturing facility.
Federal Government	BioMan	NRC	15.0	23/03/2020	NA	Funding to upgrade human therapeutics facility.
Federal Government	BioMan	NRC	29.0	23/04/2020	NA	Funding to upgrade human therapeutics facility.
Federal Government	BioMan	NRC	126.0	31/08/2020	NA	Construction of a new vaccine plant.
Western Economic Diversification	R&D	VIDO-InterVac	23.0	23/04/2020	NA	To support pre-clinical testing and clinical trials and accelerate vaccine development against COVID-19.
Atlantic Canada Opportunities	R&D	IMV	1.0	07/2020	NA	
Federal Government	R&D	Next Generation Manufacturing Cluster	2.5	05/08/2020	NA	To advance clinical development of a vaccine candidate.
Federal Government	R&D & BioMan	Next Generation Manufacturing Cluster	5.0	21/1/2021	NA	To design vaccines and expand biomanufacturing capacity.
Federal Government	R&D	NRC IRAP	150.0	01/10/2020	3	To advance the development of six COVID-19 vaccine candidates and seven therapeutics in various stages of clinical trials.
Innovative Solutions Canada	R&D	Five small & medium Firms	1.5	1/08/2020	NA	To develop COVID-19 diagnostics.
Federal Government	R&D	Data Monitoring Initiative	10.3	23/04/2020	NA	To coordinate and share pandemic-related data across Canada.
Federal Government	R&D	Canadian Immunization Research Network	10.0	23/04/2020	2	To support vaccine-related research and clinical trials, and to enhance vaccine safety and effectiveness.
Federal Government	R&D	StemCell Network	0.68	23/04/2020	NA	To support two new research projects and one clinical trial.
**Total public funding announced CAD$ million**			1406			

Abbreviations: CATCO, Canadian Treatments for COVID-19; CanCOGeN, Canadian COVID-19 Genomics Network; VIDO-InterVac, Vaccine and Infectious Disease Organization – International Vaccine Centre; NRC, National Research Council; IRAP, Industrial Research Assistance Program; WHO, World Health Organization; CIHR, Canadian Institutes of Health Research; NSERC, Natural Sciences and Engineering Research Council of Canada; R&D, Research and development; BioMan, Biomanufacturing; NA, Not Applicable; SIF, Strategic Innovation Fund. ^a^Genome Canada is a network of provincial genome centers in Canada. *Notes*: (1) Our R&D funding category includes public funding announced for research on MCMs, including antivirals, vaccines and support for clinical trials. (2) Biomanufacturing category includes public funding to upgrade existing plants or build new manufacturing facilities in the public and private sectors. (3) Some funding announced by the Government covers multiple years, ie, CAD$$ 600 million announced for SIF runs over two years and CAD$ 150 million announced for IRAP, runs over three years. Therefore, the public funding announced totals in Table 1 might not have been delivered to its beneficiaries during the time period considered in our study, and therefore may not be included in Table 2. See the definitions (Total Public Funding Announced & Public Funding Allocated) in Methods. (4) Public funding listed in Table 1 is inclusive of all funding in Table 2. Some of the lump sum funding by federal government to grant associations such as the CAD$ 600 million, and CAD$ 192 million funding to SIF, and CAD$ 150 million to NRC, are detailed in Table 2, to the extent these were distributed to beneficiaries during the timespan of our study. (5) We use “Federal Government” when the Prime Minister’s Office’s or recipient institution’s announcements do not identify the institution through which the funds would be channelled. *Source: *Authors’ compilation fromreferences: ^79-81,83-95,97-99,107,113,115,126^.

 To collect these data, we reviewed and analyzed electronically accessible project proposals, grant calls, relevant policy statements, as well as public data repositories outside of Canada such as the US Federal Securities Exchange Commission (US SEC). We used predefined search terms: COVID-19, coronavirus, funding or grants or investment or awards, and Canada (eg, COVID+Canada+grants, or COVID+Canada+funding or COVID+Canada+investment) to comprehensively locate diverse types of research funding calls for COVID-countermeasures. Data collection was complicated by the confidentiality of many funding agreements, including government’s contracts for procuring COVID-19 vaccines from several manufacturers. Although more than CAD$ 1 billion was spent in total for vaccine procurement,^78^ the precise amounts paid for each vaccine and other key terms of the agreements are not publicly available. To obtain these agreements and the terms associated with other funding sources, we filed several requests under the *Access to Information Act*^79^ with Procurement and Public Services Canada, Innovation, Science and Economic Development Canada (ISED), the CIHR, and the National Research Council (NRC) between May and June 2020. At the time of writing, only CIHR and NRC responded to our request and most of the information was redacted. We anticipate that outstanding *Access to Information Act* requests will similarly fail to result in disclosure of the agreements underpinning federal investments or grants. The procurement contracts are not incorporated into the breakdown of federal government spending due to a lack of detailed information.

 Federal funding for some grant associations runs over several years. This explains the differences between the government funding received by certain agencies, such as Strategic Innovation Fund (SIF) and Industrial Research Assistance Program (IRAP), to be allocated to the private sector (See Table 1) and the actual amounts the same agencies disbursed to the enterprises during the time period covered in this research (Table 2). To overcome the difficulties this creates for calculating respective shares of different activities within the time period of our study, we developed two distinct definitions: Public Funding Announced and Public Funding Allocated. The former relates to all public funding committed by the federal government between February 10, 2020 and March 31, 2021, including funding channelled through federal grant associations, which run over several years, and has not been disbursed to actual beneficiaries within the time period of our study. The latter includes public funding already allocated either by the Federal Government, or grant associations to actual recipients within the time period of our study. While referring to the respective shares of different purposes of public funding, such as bio manufacturing and R&D we refer to shares in allocated public funding, as their ultimate shares in announced public funding runs beyond the time period of our study.

**Table 2:** Public Funding of Private Sector Medical Countermeasures (Development of Vaccines and Therapeutics)

**Grant Institution**	**Type Funding**	**CAD$ Millions**	**Company**	**Date Announced**	**Purpose**
SIF	R&D, BioMan	175.6	AbCellera	23/03/2020	To advance antibody therapy research and construction of an antibody production facility.
SIF	R&D, BioMan	173.0	Medicago	23/10/2020	To support virus like particle vaccine and establish a large-scale vaccine and antibody production facility.
SIF	R&D	16.4	Medicago	23/03/2020	To advance clinical trials of its plant-based candidate vaccine.
SIF	R&D	56.0	Variation Biotechnologies	05/08/2020	To support clinical trials for a COVID-related vaccine candidate.
SIF	R&D	18.2	Precision Nano Systems Inc.	23/10/2020	To support development a COVID-19 vaccine candidate through preclinical studies and clinical trials.
SIF	R&D	25.1	Precision Nano Systems Inc.	02/ 02/2020	To expand capabilities in the production of ribonucleic acid vaccines and genetic medicines.
SIF	R&D	6.7	Arch Biopartners	15/12/2020	To advance drug candidate to treat organ inflammation through Phase II clinical trials.
SIF	R&D	13.4	Immune Biosolutions	16/03/2021	To advance therapeutic candidates from preclinical studies to Phase II clinical trials.
SIF	R&D	14.0	Edesa Biotech	02/2/2021	To develop a monoclonal antibody therapy (EB05).
SIF	BioMan	32.7	Novocol	16/03/2021	To expand manufacturing facilities.
SIF	BioMan	54.2	Kabs Laboratories	16/03/2021	To expand biomanufacturing capacity.
Next Generation Manufacturing Supercluster	R&D, BioMan	5.0	Providence Therapeutics & Northern RNA Inc.	21/01/2021	To design COVID-19 vaccines and expand vaccine manufacturing capacity.
Next Generation Manufacturing Supercluster	R&D	2.5	IMV	5/08/2020	R&D to advance clinical development of vaccine candidate.
Atlantic Canada Opportunities	R&D	1.0	IMV	07/2020	To develop COVID-19 vaccine candidate.
NRC – IRAP	R&D	5.4	IMV	08/10/2020	To support the continuation of clinical trials for IMV’s DPX-COVID-19 vaccine candidate.
NRC – IRAP	R&D	5.0	Entos Pharmaceuticals	23/10/2020	To advance COVID-19 DNA vaccine to phase I human clinical trials.
NRC – IRAP	R&D	4.7	Providence Therapeutics	27/10/2020	To support phase 1 clinical trials of its promising and proprietary mRNA COVID-19 vaccine.
NRC – IRAP	R&D	1.9	HyperMabs	18/12/2020	To support the development of FB100 therapeutic.
NRC – IRAP	R&D	1.7	Mannin Research	18/12/2020	To support development of a COVID-19 therapeutic.
NRC – IRAP	R&D	4.0	Glycovax Pharma	23/10/2020	Phase 1 clinical trials of its COVID-19 vaccine.
NRC – IRAP	R&D	2.8	Symvivo	23/10/2020	To support the clinical advancement of oral, DNA vaccine candidate.
NRC – IRAP	R&D	1.3	Biodextris Inc.	23/10/2020	For preclinical development of a nasal COVID-19 vaccine candidate.
NRC – IRAP	R&D	0.3	Bold Therapeutics	18/12/2020	To prepare preclinical efficacy data, and support for clinical trials.
NRC – IRAP	R&D	4.6	JN Nova Pharma	18/12/2020	To assist the development of a proprietary drug.
NRC – IRAP	R&D	4.2	Laurent Pharmaceuticals	18/12/2020	For clinical development of LAU-7b antiviral and inflammation controlling therapy.
NRC – IRAP	R&D	0.1	QU Biologics	18/12/2020	To provide proof-of-concept evidence for the safety and efficacy of a preventative treatment.
NRC – IRAP	R&D	1.2	Vasomune Therapeutics Inc.	18/12/2020	To support phase 1 clinical trials of its AV-001 drug candidate.
Innovative Solutions Canada	R&D	0.3	Galenvs	10/2020	To adapt magnetic based reagents for RNA extraction.
Innovative Solutions Canada	R&D	0.3	Deep Biologics	1/8/2020	To develop palm size device to detect SARS-CoV-2.
Innovative Solutions Canada	R&D	0.3	Fourien	1/8/2020	Disposable point of care device for rapid detection of SARS-CoV-2.
Innovative Solutions Canada	R&D	0.3	Metabolic Insights	1/8/2020	To Adapt device to detect viral protein of SARS-CoV-2.
Innovative Solutions Canada	R&D	0.3	Nicoya Lifesciences	1/8/2020	To develop rapid device to detect viral protein of SARS CoV-2.
**Public funding allocated for private sector (CAD$ millions)**		633			

Abbreviations: NRC, National Research Council; IRAP, Industrial Research Assistance Program; R&D, Research and development; SIF, Strategic Innovation Fund; BioMan, Biomanufacturing. Notes: (1) CAD$ 37 million out of CAD$ 150 million funding announced for NRC IRAP funding was disbursed for the early stage development of six vaccines and seven therapeutic candidates. The remainder 113 million was spared for the development of phase II stages of the most successful candidates from amongst those funded. 2) CAD$ 16.4 million R&D public funding figure is an estimate calculated by subtracting CAD$ 175.6 million awarded to AbCellera from CAD$ 192 million announced as awarded to two companies on March 23, 2020. Source: Authors’ compilation from references: ^79-81,83-99,107,113,115^. All operations ceased for Medicago Inc. as of February 3, 2023 (https://medicago.com/en/press-release/cessation-of-operations-at-medicago/).

 A more detailed explanation of the data collection, extraction and triangulation methods as well as our definitions and categorization of public funding is included in Supplementary file 1, Data Collection Methods for Public Funding of MCMs in Canada.

###  Results: Public Funding of Medical Countermeasures by the Canadian Government 

 During the first year of the pandemic (February 10, 2020 to March 31, 2021), the Government of Canada (GoC) announced CAD$ 1.4 billion in funding for the research, development and manufacturing of COVID-19 MCMs (See Table 1). CAD$ 1 billion of this was allocated to the recipients within the period covered under our study, with the remaining to be distributed over 2022-2023.

 The public funding was distributed through well-established channels such as CIHR and NRC as well as through newer mechanisms such as the SIF and regional development programs such as the Western Economic Diversification, organized under ISED. Funding was also directly provided to public-private-non-profit research platforms, such as Stem Cell Network, Genome Canada, and the Canadian Immunization Research Network^80^ (Table 1).

 The largest portion of the announced federal funding (56%, roughly CAD$ 792 million) was channelled through ISED’s SIF towards large-scale investment in private sector firms such as AbCellera, Medicago, Precision Nanosystems, and Variation Biotechnologies (Table 2 identifies the purpose of each SIF grant).

 The second highest share of funding (23%, CAD$ 320 million) was channelled through Canada’s largest R&D organization,^81^ the NRC. The first three rounds were invested in the NRC’s biomanufacturing capacity, upgrading existing plants ($15 million and $29 million respectively to upgrade the Human Health Therapeutics plant)^80,82,83^ and constructing a new Biologics Manufacturing Centre ($126 million) in Montreal to secure capacity for an estimated 2 million doses of vaccines per month, compliant with Good Manufacturing Practices standards.^84^ In contrast, the fourth round of funding (CAD$ 150 million) in October 2020 was distributed outside the NRC, through its longstanding IRAP. IRAP has contributed CAD$ 23 million for early stage R&D of 6 vaccine candidates^85^ and CAD$ 13.9 million for seven therapeutics being developed by small and medium-sized firms across Canada^86^ (See Table 2). The remaining $113 million funding was spared to advance the most promising recipients of this initial funding to later stages.^87^

 Constituting 12% (CAD$ 175.4 million) of the total public funding announced, research grants were channelled through the Tri-Agency Council, primarily CIHR — Canada’s largest funder of health research. Two rounds of CIHR grant competitions, held in February/March and May 2020 with a total budget of CAD$ 166 million funded 240 research projects for MCMs as well as social and policy interventions. In each competition, biomedical research dominated both the number and amount, securing 60% of the total number of awarded projects and 75% of available funding. Amongst MCMs, COVID-19 therapeutic development projects received the highest share of CIHR funding in both numbers (33%) and amounts (37%). Clinical management of COVID-19 came second at 30% of projects funded while diagnostic development received 20%. Vaccine development received the least amount of CIHR funding.^88-94^

 The largest portion of federal funding announced (68% of the total and roughly CAD$ 959 million) was channelled towards investment in private sector firms through SIF, NRC IRAP, and other funding streams. Federal investments in the private sector focused on enhancing manufacturing, R&D capacity of small and medium-sized (defined according to the number of paid employees) health and biosciences companies based in Canada (Table S1 in Supplementary file 2).

 25% of the federal funding allocated (CAD$ 269 million) was invested exclusively in restoring Canada’s biomanufacturing capacity.^95^ 43% of funding allocated (CAD$ 463 million) was invested in research to develop COVID-19 MCMs outside of manufacturing. The remaining 33% of public funding (CAD$ 354 million) was allocated to private sector for both manufacturing and R&D purposes (including CAD$ 175.6 million to AbCellera, $173 million to Medicago and CAD$ 5 million to Providence). ISED data do not provide breakdown of this combined private sector funding for biomanufacturing and R&D purposes.

 Federal investments to scale up biomanufacturing capacity were directed to both public (eg, University of Saskatchewan’s The Vaccine and Infectious Disease Organization – International Vaccine Centre [VIDO-InterVac], NRC) and private (eg, Medicago, AbCellera, Precision Nano Sytems) sectors.^96^ The largest public investment in a private corporation during the pandemic was the Federal Government’s CAD$ 415 million contribution to Sanofi Pasteur’s vaccine manufacturing plant in Toronto.^87^ As this investment concerned end-to-end influenza vaccine manufacturing and was not a direct COVID-19 MCM, it was not included in our COVID-19 public funding calculations. In total, within the time period under consideration in our study, the federal government invested in nine biomanufacturing projects (excluding the Sanofi plant), with only one, the NRC plant in Montreal completed within the anticipated duration of COVID-19 pandemic, in June 2021. Timeframes for other projects extend several years ahead.^97^

 In addition to funding allocated for the development of MCMs by private and public actors within Canada’s borders, the federal government invested more than CAD$ 1 billion in procurement contracts (also known as “Advance Market Commitments”) to seven multinational vaccine manufacturers, with options to secure a total of up to 402 m doses. These include Pfizer: up to 76 million, Astra Zeneca: 20 million, Johnson & Johnson: up to 38 million, Medicago: up to 76 million, Moderna: 44 million, Novavax: up to 76 million, Sanofi/GSK: 72 million.^84,98,99^ As we detail below, these contracts only became publicly available in June 2021, and were heavily redacted.

 Canada also invested CAD$ 220 million in the COVAX initiative^92^ to procure 15 million doses of COVID-19 vaccines through COVAX. Canada donated an additional CAD$ 325 million to the COVAX Advance Market Commitment Mechanism to subsidize vaccine purchases by low-income countries.^98,100^

 Federal Government’s COVID-19 MCMs funding and procurement decisions were guided by three external advisory bodies, appointed during the early days of the pandemic. A Vaccine Task Force advised the government about the development of domestic vaccine candidates and procurement of international vaccine candidates. Therapeutics Task Force advised the selection of COVID-19 therapeutics eligible for public funding. A joint Biomanufacturing Subcommittee consisting of members of the Therapeutics and Vaccine Task Forces advised on public investments to expand manufacturing capacity in the biopharmaceuticals sector. Consisting of academics, researchers, and private sector representatives including biopharmaceutical firms, venture capital firms, several members of external advisory bodies have considerable potential for scientific and financial conflicts of interest.^101-104^

 One of the hallmarks of public funding during the pandemic was the opacity of terms and conditions of decision-making processes surrounding public funding. For instance, no publicly available information exists about the appointment criteria for the Task Forces that made the most important funding decisions. Although Task Force meetings included a Declaration of Interests Protocol that required members with conflicts of interests to recuse themselves from discussing recommendations,^105^ there are no publicly available records of their work, including meeting reports, and texts outlining funding criteria used to select proposals.

 Unlike the United States, where Operation Warp Speed research contracts are posted on the Department of Health and Human Services’s website,^106^ in Canada none of the contracts disbursed by federal funding institutions, such as the SIF, are made public. No information is available in the public domain about funding conditions, project work or public safeguards. The same is true about the government’s public procurement initiatives. While some limited and heavily redacted information is released about the public procurement of medical supplies, the contracts have not been made public. Procurement and research contracts are available through Access to Information requests but the processing of such requests not only takes longer during the pandemic, but when provided their content is heavily redacted.

 The government did not publish the vaccine procurement contracts with transnational biopharmaceutical firms until it was compelled to do so in a minority Parliament. When an October 26, 2020 Parliamentary Order required the disclosure of contracts along with other COVID-19 response documents, both Canada’s Public Services and Procurement Minister and Pfizer Canada warned that the measure would harm arrangements with manufacturers of MCM and the ongoing talks to secure additional supply.^107^ The contracts were not released until June 11, 2021, four months after a second official request issued by House Health Committee, and then only after extensive redactions carried out at the Public Services and Procurement.^108^ As seen from Table S2 in the Supplementary file 3, redacted versions of vaccine contracts lack all crucial details, such as delivery dates, prices paid, or IP clauses that would allow meaningful insights into protection of public interest.

 Early in the pandemic, both globally and in Canada, there were motions for a changing approach to public funding to facilitate an open sharing of knowledge. The GoC, as well as granting institutions, such as the CIHR, Genome Canada, and NRC signed the Wellcome Trust’s Joint Statement on Sharing Research Data and Findings relevant to the novel coronavirus outbreak^109^ which called on researchers, journals, and funders to ensure that research findings, data, peer-reviewed publications resulting from research relevant to COVID-19 were shared rapidly, openly, and freely to inform public health response. The new compulsory licensing measures that Canada enacted in Parliament, on March 25, 2020, as part of *the COVID-19 Emergency Response Act* endorsed an active approach to ensure the availability of COVID-19 MCMs, and the protection of public interest.^15^ The measure provided more comprehensive powers to the government than were available in Canada’s existing compulsory licensing provisions^110^ allowing the government to immediately license a vaccine or drug without first consulting the patent-holders, and determining the appropriate compensation only after use. Yet, Canada’s new compulsory licensing measures were time-restricted and when no action was taken to renew them by the government, they lapsed on September 30, 2020. Hence, during the remainder of the pandemic, public funding policy upheld an IP intensive approach to knowledge production.

 The Canadian government also did not operationalize its export oriented compulsory licensing scheme — Canada Access to Medicines Regime (CAMR) — to facilitate access to COVID-19 vaccines in low-income countries. Legislated in 2004, after the famous international compromise reached at TRIPS Doha Declaration, which introduced exceptions to patent rights hindering exports of patented products produced under a compulsory license, CAMR allows Canadian generic manufacturers to produce and export patented products to countries that lack manufacturing capacity.^111,112^ When a Canadian company with domestic manufacturing capacity, Biolyse, lobbied to trigger the CAMR’s export-oriented compulsory licensing option, to produce Johnson and Johnson’s COVID-19 vaccine, the government blocked such efforts by creating new regulatory barriers for the manufacturer.^112^

 Canadian public funding institutions conveyed a pro IP approach, stating “the default position of Canada is to allow funding recipients to retain the IP rights associated with their solutions.”^113^ CIHR emphasized that the GoC did not own the IP arising from the work and it cannot directly benefit from research outputs.^114^ Although the CIHR Calls warned that Rapid Response and Rapid Research might have special provisions, CIHR’s response to our ATI request showed that the Terms and Conditions sections of the contracts did not contain any IP rights provisions that were specific to COVID-19.^115^

 It is not possible to make a comprehensive assessment of IP management in public funding contracts, as the government’s research contracts are still not public. Two of the SIF funding agreements, AbCellera^116^ and Variation Technologies, or Variation Biotechnologies Inc. (VBI) Vaccines, the name of its parent company in Massachusetts, US^117^ became available via the US SEC database. AbCellera received a repayable CAD$ 175.6 million from SIF whereas VBI was awarded a non-repayable CAD$ 56 CAD million. Despite the difference, there are some similarities between the two agreements. For example, Articles 6.3.3 and 6.5 of AbCellera contract and Articles 6.3.4, and 6.4 of VBI contract both seek to impose obligations on the recipients to maintain work, create employment, provide training opportunities, and conduct clinical trials in Canada. Articles 6.3.2 and 11.2 in AbCellera, and Articles 11.2, 11.4, and 11.7 in VBI contracts require the ownership of project IP by the recipients of funds, necessitate the Minister of ISED’s permission before granting exclusive licenses to third parties, and necessitate the creation and retention of IP in Canada. Finally, Article 6.3.1 in both AbCellera and VBI contracts include requirements to make products “accessible and available to Canadians.”

 Beyond those conditions, the two contracts and accompanying contexts differ markedly. The VBI contract contains several additional safeguards pertaining to product availability and affordability. Article 6.3.1 of the VBI contract entitled “Strengthen Canada’s capability to respond to COVID-19 and future pandemic”supplements the accessibility and availability requirement noted in Article 6.3 of AbCellera contract with a requirement for timeliness, and includes a commitment to increase not only domesticbut also global availability and affordability of any resulting vaccines. Entitled “Project Intellectual Property Use in Response to COVID-19,”Article 11.8 of the VBI contract allows the government to use (*i*) the Project Intellectual Property and Project Intellectual Property Rights; and (*ii*) Background Intellectual Property and Background Intellectual Property Rights owned by the Recipient or, licensed by the Recipient if the company fails to ensure a sufficient domestically-sourced supply of vaccines in response to COVID-19. Broadly interpreted, this clause appears to allow the government to override any patent rights associated with VBI’s products as well as the manufacturing know-how. In contrast, none of these added access measures are present in the AbCellera contract.

 Despite Canadian public funding institutions’ support for Wellcome Trust’s Statement on Sharing Research Data and Findings, clinical trial data from publicly funded research grants were not shared widely. Out of 144 beneficiaries that received funding for the development of MCMs in CIHR’s largest funding stream, Rapid Research and Rapid Response, our research retrieved only two registry entries in clinical trials.gov, the largest and most popular platform for public disclosure of clinical trial results.

## Discussion

 Public funding has been the most widely deployed intervention to mobilize the development of MCMs to help fight COVID-19 in Canada other advanced economies, such as the United States and EU.^118^ Extensive public funding for COVID-19 research is warranted, as are calls for fair, just, and transparent access to the resulting knowledge and products. Funding contracts that include upfront commitments from beneficiaries to safeguard public access to affordable, transparent and sustainable research outputs^119^ can ensure that public funding of scientific research conforms to principles of democratic accountability. As our background review showed, imperatives imposed by financialization, such as market-based innovation policies, IP intensive knowledge production, and the erosion of local manufacturing policies, make it harder for governments to negotiate such policies. Nevertheless, in the wake of twin public health and economic crises spurred by the COVID-19 pandemic, governments both in Canada and abroad made statements to help protect public interest when they were funding MCM development. In this section, we discuss whether practices and priorities underpinning Canada’s public funding of COVID-19 research keep these commitments, and whether this funding of MCMs marks a shift from established practices of financialization.

 Ruptures in global supply chains experienced during the COVID-19 pandemic highlighted the strategic importance of domestic biopharmaceutical manufacturing capabilities to both governments and international organizations. Recognizing the limited state of Canada’s domestic manufacturing capabilities,^7^ the government channelled a significant proportion of its COVID-19 related public funding into the development and scale-up of domestic manufacturing capabilities.

 The government’s choice of investing in domestic manufacturing may appear as a rift in neoliberal orthodoxy that discourages direct public investment. However, even though the government invested in existing public manufacturing capacity, policy solutions prioritized private sector involvement rather than public ownership or management of manufacturing facilities. For instance, the cost of NRC’s CAD$ 126 million new biomanufacturing site was covered by federal funding, and despite no available private partners the investment was announced as a public private partnership.^120^ Approximately one month later, the government convinced a transnational vaccine developer, Novavax, to produce its vaccines at NRC’s new facilities, with construction completed in June 2021.^121,122^ Despite calls by health experts and opposition groups for a publicly owned vaccine plant,^123,124^ the government selected a public private partnership model for the management of public investments in domestic manufacturing.^7^

 Although the CAD$ 415 million federal investment to expand the capacity of Sanofi’s vaccine plant in Toronto was not a direct COVID-19 measure^125^ the government’s adherence to market based solutions, is indicative of priorities underpinning public funding policy. While the same facility was once Canada’s publicly governed Connaught Laboratories before it was privatized in the 1980s,^126^ there is no indication that the government’s investment carries any obligations upon Sanofi to seek outside input into what, when, and how vaccines will be produced. Unless it is disclosed under the US securities law, the Sanofi agreement, as per the norms of financialization, is likely to remain strictly confidential — another proprietary asset that is part and parcel of an IP-intensive approach still dominating biopharmaceutical knowledge governance.

 In Canada, the extensive public funding of COVID-19 products was accompanied by neither consistent nor coherent access policies to balance IP exclusivities with public safeguards. A prime example was rendering useless of new compulsory licensing measures added to the Patent Act early in March 25, 2020 due to the lack of political will to extend the 6 months deadline on the measure. The Canadian government’s preference for not operationalizing its export oriented compulsory licensing scheme — CAMR — to facilitate access to COVID-19 vaccines in low-income countries is also compatible with its IP intensive approach in public funding.

 The government also chose not to leverage its extensive funding initiatives by integrating access commitments into funded research grants. While the Canadian grant institutions’ signing onto the Wellcome Trust’s Joint Statement on Sharing Research Data and Findings is an important initiative, its scope remains limited. The statement does not specify public disclosure of clinical data and its commitments towards data transparency are too broad to ensure the protection of public health and further scientific inquiry.

 Our analysis of SIF contracts that became available via the US SEC database reveals a bespoke, and in one case ineffective, approach to access related concerns. While the VBI contract contains several additional safeguards pertaining to product availability and affordability, none of the added access conditions are present in the AbCellera contract. This is likely because shortly before signing the SIF contract, AbCellera entered into exclusive licensing of its technology to pharmaceutical giant Eli Lily to further develop and manufacture its monoclonal antibody therapy bamlanivimab.^127^ With control over the IP already transferred to the multinational company, the access measures seemingly built into the federal government’s CAD$ 175,6 million investment in AbCellera are thus reduced to maintaining AbCellera’s platform technology in Canada and the construction of its biomanufacturing plant.

 With no other SIF contracts publicly available, it is impossible to discern whether the terms and conditions of the VBI agreement versus the AbCellera agreement better reflect the government’s preferred approach. The norms of biopharmaceutical financialization, and attendant prioritization of IP protection, and reliance upon globalized and privatized supply chains, coupled with the federal government’s demonstrated adherence to business-as-usual with respect to its other spending mechanisms, suggest that the VBI agreement is an exception that proves the rule.

 The limited amount of information available about the funding process, decision-making procedures and purposes for which the funding is put seems to be a hallmark feature of the public funding of COVID-19 MCMs in Canada. The opacity surrounding public funding decision-making processes was strategically used for preventing the assessment of the government’s public funding of COVID-19 MCMs. Such opacity also highlights the features of financialization that undermine tax payer rights and democratic accountability.^128^

 Our findings about the public funding of COVID-19 MCMs during the pandemic resonate with research from other high-income countries.^18,129^ For instance, public funding in the United States, and the United Kingdom also focussed on expanding domestic manufacturing capacity and did not compel nor encouraged beneficiaries to share their technology.^18^ Similar to patterns observed in Canada, public funding in the United States, United Kingdom, and Germany focussed on downstream research, and the development of COVID-19 MCMs. Finally, although there is great diversity in policies to foster public interest across different countries, the opacity of public funding,^16,18,130^ and negotiations between governments and the biopharmaceutical industry during the pandemic have been observed as a common concern in all high-income economies. Transparency International^131^ reports that of 182 agreements for the purchase of 12 different COVID-19 vaccines by 75 buyers (mostly governments and multilateral organizations) and suppliers (biopharmaceutical firms), only 11 were formally published. Out of 11 formally published contracts, 10 were heavily redacted.

## Conclusion

 Developments spurred by financialization in the biopharmaceuticals sector undermined public access to MCMs during the COVID-19 pandemic. Our analysis highlights that there has been some reorientation in public policy goals, reflected in rising awareness about the need to create domestic biomanufacturing capabilities. Beyond that incremental change, Canada’s public funding of MCMs during the COVID-19 pandemic shows no apparent structural change from broader established practices of financialization in the biopharmaceutical industry. Public funding of research, development and manufacturing of MCMs continue to be structured in ways that prioritize the primacy of market based choices and profit-maximizing measures as the superior policy axioms. This approach is also reflected in the IP management of public funding, which tends to refrain from balancing exclusivities with access requirements. Another long-lasting feature in public funding policies during the pandemic has been the opacity that surrounds decision-making processes about public funding. The government’s strict adherence to confidentiality about its public funding and expenditures breaches a fundamental requirement of democratic government. Given the large amount of public funding, as well as the importance of accessible MCMs to address current and future pandemics, the government’s continued sanctioning of secrecy – in and of itself – signals financialized capitalism’s stranglehold on biopharmaceutical innovation.

## Acknowledgements

 We would like to thank Andrea MacGregor for her assistance in data compilation during the early stages of the research for this article. We would also like to thank Katherine Fierlbeck for her feedback on earlier drafts of our manuscript.

## Ethical issues

 The research received approval from Dalhousie University, Health Sciences Research Ethics Board (REB 2019-4953).

## Competing interests

 MH reported being a member of the Patented Medicine Prices Review Board, Canada’s national drug price regulator, and receiving honoraria from the Board for his service.

## Funding

 This work was supported by research funding from Canadian Institutes of Health Research (CIHR PJT 156256).

## Supplementary files


Supplementary file 1. Data Collection Methods for Public Funding of MCMs in Canada.^95,132^

Supplementary file 2 contains Table S1.

Supplementary file 3 contains Table S2.
