# Supplementary material for: Biopharmaceutical Financialization and Public Funding of Medical Countermeasures (MCMs) in Canada During the COVID-19 Pandemic
Source: Int J Health Policy Manag. 2023 May 27;12:6936. doi: 10.34172/ijhpm.2023.6936 (PMC10462233; doi:10.34172/ijhpm.2023.6936)
Supplement: Supplementary file 1 — Data Collection Methods for Public Funding of MCMs in Canada. 95,132 [file ijhpm-12-6936-s001.pdf]

Article title: Biopharmaceutical Financialization and Public Funding of Medical Countermeasures (MCMs) in Canada During the COVID-19 Pandemic

Journal name: International Journal of Health Policy and Management (IJHPM)

Authors' information: Ipek Eren Vural[1,2,3]* , Matthew Herder[4,5] , Agnieszka Doll[5,6] , Janice E. Graham[7,8]

[1]Department of Political Science & Public Administration, Middle East Technical University, Ankara, Turkey.

[2]Department of Political Science, Dalhousie University, Halifax, NS, Canada.

[3]Faculty of Health Sciences, Simon Fraser University, Burnaby, BC, Canada.

[4]Health Law Institute, Dalhousie University, Halifax, NS, Canada.

[5]Department of Pharmacology, Dalhousie University, Halifax, NS, Canada.

[6]Department of History and Sociology, University of British Columbia Okanagan, BC, Canada.

[7]Department of Pediatrics, Dalhousie University, Halifax, NS, Canada.

[8]Department of Sociology and Social Anthropology, Dalhousie University, Halifax, NS, Canada.

(*Corresponding author: IVural@dal.ca)

Supplementary file 1. Data Collection Methods for Public Funding of MCMs in Canada.

Data collection consisted of two stages. In the first stage, two researchers, IEV and AD identified and mapped institutions, grant policies, and calls for publicly funded COVID-19 research and the Canadian Government's announcements. Early data were collected through two Internet searches conducted during March 27-April 9, 2020, and April 15 – May 15, 2020, for publicly funded COVID-19 research calls in Canada. From September 2020 to March 31, 2021, IEV completed the data collection process, updating the search for public funding and grant calls quarterly.

COVID-19 funding calls information retrieved from internet search with predefined terms was followed to the original website of the federal grant agency or the government to retrieve complete information about the types of funding and their conditions. Other publicly available funding trackers, including:

https://launchandscalefaster.org/COVID-19

https://www.knowledgeportalia.org/covid19-r-d-funding,

https://www.policycuresresearch.org/covid-19-r-d-tracker/,

https://www.infoedglobal.com/COVID19/COVID-19,

https:// covid19resources.ca/fundedresearch.html

was searched to sporadically to triangulate our research on public funding announcements. Data from government documents, announcements, reports, news in print and online media were also included in the analysis to identify funding narratives, principles, goals, conditions and amounts.

U.S. Securities and Exchange Commission filings submitted by listed private companies were searched to retrieve some of the government contracts by companies receiving public funding in Canada. Health Canada's Clinical Trials database, (https://www.canada.ca/en/health-canada/services/drugs-health-products/drug-products/health-canada-clinical-trials-database.html) and ClinicalTrials.gov (maintained by National Library of Medicine) were used to check the number of registrations by CIHR funding recipients. Subscription based databases such as Mergent, Factiva, Unilex were also searched to retrieve company-based information about the private sector firms that received public funding. Public funding information retrieved was used to build a database that details the conditions and terms of each funding.

Access to Information requests was filed in May 2020 with major governmental institutions managing public funding, including the CIHR (A-2020-0010), National Research Council, NRC (A2020-0005), Innovation Science and Economic Development Canada (ISED), Ministry of Public Services, and Procurement (2020_007547), to access research, grant, and government contracts and analyze funding terms. From each federal agency, we requested research grant agreements, contracts, government records, letters of intent, or other memoranda of understanding that may have been entered between the federal government and a partner (whether public or private) related to COVID-19 and outline the specific requirements and other terms associated with such funding, including the conditions of funding attached to each grant, award or contract. Only CIHR and NRC responded to our ATI requests.

During the study design, the authors planned to follow public funding for the duration of one year, starting from the onset of funding disbursements in Canada on February 10, 2020. The authors agreed to extend the observation for a period maximum of six months in case of extraordinary developments in the funding process. On March 31, 2021, the Canadian government disbursed the largest lump sum amount to a private corporation during its COVID-19 funding. Accordingly, the researchers decided to include March 31, 2021 as the cut point.

Funding agencies' categorizations for the type, purpose and domains of public funding was adopted whenever available and triangulated against other sources. For example, most public funding agencies identified the type of funding provided in their press releases. Research grants were used to refer to public funding provided by federal grant associations such as CIHR, NSERC towards mostly university based research and development projects of MCMs. Public Private partnerships involved joint investments by federal, private and non profit organizations, with no specification about management conditions. Direct funding was used to refer to funding disbursed through government contracts to public or private bodies for specific purposes.

CIHR's categorization and figures were used for the analysis and distribution of its funding across medical and social and policy countermeasures, as well as subcategories under each countermeasure. Likewise, ISED's categorization of funding purposes in its backgrounder documents [95, 132] was used to calculate total amounts invested by the Strategic Innovation Fund (SIF). ISED data did not include the breakdown of public funding to private companies when such funding combined research and development with manufacturing. This led to difficulty in calculating exact amounts used for research, development, and manufacturing of COVID 19 medical countermeasures. We therefore referred to funding to private sector that combined research, development and manufacturing as a separate category.

Finally, while analyzing the size composition of private sector recipients of public funding, we used Statistics Canada's definition of small and medium sized companies, which considers a small enterprise as one with fewer than 100 employees, a medium-sized enterprise as one with 100 to 499 employees, and a large enterprise as one with over 500 employees.
