# Supplementary material for: Biopharmaceutical Financialization and Public Funding of Medical Countermeasures (MCMs) in Canada During the COVID-19 Pandemic
Source: Int J Health Policy Manag. 2023 May 27;12:6936. doi: 10.34172/ijhpm.2023.6936 (PMC10462233; doi:10.34172/ijhpm.2023.6936)
Supplement: Supplementary file 2 — contains Table S1. [file ijhpm-12-6936-s002.pdf]

Article title: Biopharmaceutical Financialization and Public Funding of Medical Countermeasures (MCMs) in Canada During the COVID-19 Pandemic

Journal name: International Journal of Health Policy and Management (IJHPM)

Authors' information: Ipek Eren Vural[1,2,3]*, Matthew Herder[4,5], Agnieszka Doll[5,6], Janice E. Graham[7,8]

[1]Department of Political Science & Public Administration, Middle East Technical University, Ankara, Turkey.

[2]Department of Political Science, Dalhousie University, Halifax, NS, Canada.

[3]Faculty of Health Sciences, Simon Fraser University, Burnaby, BC, Canada.

[4]Health Law Institute, Dalhousie University, Halifax, NS, Canada.

[5]Department of Pharmacology, Dalhousie University, Halifax, NS, Canada.

[6]Department of History and Sociology, University of British Columbia Okanagan, BC, Canada.

[7]Department of Pediatrics, Dalhousie University, Halifax, NS, Canada.

[8]Department of Sociology and Social Anthropology, Dalhousie University, Halifax, NS, Canada.

(*Corresponding author: IVural@dal.ca)

Supplementary file 2

Table S1. Information on Private Sector Companies that received Public Funding

**Company**	**No of Paid Employees**	**Revenues (CAD $ Million)**
AbCellera Biologics	386	507
Medicago	204	NA
Variation Biotechnologies Inc.	149	1
Precision Nano Systems	80	20

Inc.		
Arch Biopartners	13	4
Sanofi Pasteur Limited	1100	NA
Novocol Pharmaceutical of Canada	196	38.2
KABS Labaratories Inc.	147	30.4
Edesa Biotech	16	NA
Immune Biosolutions	34	6.4
IMV	97	0.18
Entos Pharmaceuticals	24	8.1
Providence Therapeutics	30	6
HyperMabs Inc.	30	33
Mannin Research Inc.	6	2.3
Glycovax Pharma	11	2.9
Symvivo	8	1.4
Biodextris Inc	11	2
Bold Therapeutics	20	3.8
JN Nova Pharma	NA	NA
Laurent Pharmaceuticals	6	0.9
QU Biologics	16	4.2
Vasomune Therapeutiics Inc	27	5.1
Galenvs	20	4.5
Deep Biologics	5	0.8
Fourrien	16	4
Metabolic Insights	NA	NA
Nicoya Lifesciences	50	10.5

Notes Table S.1: 1. Statistics Canada considers a small enterprise as one with fewer than 100 employees, including those that do not report any employment. Medium-sized enterprises have 100 to 499 employees, and a large one has over 500.
2. NA: Not Available.
