# Supplementary material for: Biopharmaceutical Financialization and Public Funding of Medical Countermeasures (MCMs) in Canada During the COVID-19 Pandemic
Source: Int J Health Policy Manag. 2023 May 27;12:6936. doi: 10.34172/ijhpm.2023.6936 (PMC10462233; doi:10.34172/ijhpm.2023.6936)
Supplement: Supplementary file 3 — contains Table S2. [file ijhpm-12-6936-s003.pdf]

Article title: Biopharmaceutical Financialization and Public Funding of Medical Countermeasures (MCMs) in Canada During the COVID-19 Pandemic

Journal name: International Journal of Health Policy and Management (IJHPM)

Authors' information: Ipek Eren Vural[1,2,3]* , Matthew Herder[4,5] , Agnieszka Doll[5,6] , Janice E. Graham[7,8]

[1]Department of Political Science & Public Administration, Middle East Technical University, Ankara, Turkey.

[2]Department of Political Science, Dalhousie University, Halifax, NS, Canada.

[3]Faculty of Health Sciences, Simon Fraser University, Burnaby, BC, Canada.

[4]Health Law Institute, Dalhousie University, Halifax, NS, Canada.

[5]Department of Pharmacology, Dalhousie University, Halifax, NS, Canada.

[6]Department of History and Sociology, University of British Columbia Okanagan, BC, Canada.

[7]Department of Pediatrics, Dalhousie University, Halifax, NS, Canada.

[8]Department of Sociology and Social Anthropology, Dalhousie University, Halifax, NS, Canada.

(*Corresponding author: IVural@dal.ca)

Supplementary file 3

Table S2. Information on Vaccine Contracts concluded by the Canadian Government

Contract Information of Public Interest Relevance	SANOFI	MEDICAGO	ASTRAZENECA	MODERNA	PFIZER	NOVAVAX	JOHNSON & JOHNSON
Contract Value	R	R	R	R	N	R	R
Quantities	Y	Y	Y	Y	Y	Y	Y
Timetable for Delivery	R	R	R	R	R	R	R
Price per Vaccine Dose	R	R	R	R	R	R	R
Ability to donate excess doses	Y	Y	Y	Y	N	Y	Y

Ownership rights to develop	Y	Y	Y	Y	N	N	Y
March in Rights	N	N	N	N	N	N	R
IPR	R	R	R	R	R	N	R
Penalties for non Performance	R	R	R	Y	R	R	R
Scope and Duration of Indemnification	R	R	R	R	N	Y	N

Authors’ compilation. Criteria of public interest sought in the vaccine contracts draw on Transparency International, 2021.
N: No. The information does not exist in the agreement, it is not certain whether it is redacted or not covered at all.
R: The information is redacted in the released contract.
Y: Yes. The Information exists in the released contrac.
